# Molecular mechanisms of long noncoding RNAs and their role in disease pathogenesis

**DOI:** 10.18632/oncotarget.24307

**Published:** 2018-01-01

**Authors:** Guoku Hu, Fang Niu, Bree A. Humburg, Ke Liao, Sunil Bendi, Shannon Callen, Howard S. Fox, Shilpa Buch

**Affiliations:** ^1^ Department of Pharmacology and Experimental Neuroscience, University of Nebraska Medical Center, Omaha, NE, USA

**Keywords:** long non-coding RNA, CNS disorder, cancer, therapy

## Abstract

LncRNAs are long non-coding regulatory RNAs that are longer than 200 nucleotides. One of the major functions of lncRNAs is the regulation of specific gene expression at multiple steps including, recruitment and expression of basal transcription machinery, post-transcriptional modifications and epigenetics [1]. Emerging evidence suggests that lncRNAs also play a critical role in maintaining tissue homeostasis during physiological and pathological conditions, lipid homeostasis, as well as epithelial and smooth muscle cell homeostasis, a topic that has been elegantly reviewed [2–5]. While aberrant expression of lncRNAs has been implicated in several disease conditions, there is paucity of information about their contribution to the etiology of diseases [6]. Several studies have compared the expression of lncRNAs under normal and cancerous conditions and found differential expression of several lncRNAs, suggesting thereby an involvement of lncRNAs in disease processes [7, 8]. Furthermore, the ability of lncRNAs to influence epigenetic changes also underlies their role in disease pathogenesis since epigenetic regulation is known to play a critical role in many human diseases [1]. LncRNAs thus are not only involved in homeostatic functioning but also play a vital role in the progression of many diseases, thereby underscoring their potential as novel therapeutic targets for the alleviation of a variety of human disease conditions.

## INTRODUCTION

Long non-coding RNAs (lncRNAs) are a large and diverse class of transcribed RNA molecules that lack many signature motifs of mRNAs, including the Kozak consensus sequence [9, 10] and open reading frame (ORF) of significant lengths [11]. To date it has been estimated that the human genome contains approximately 51,382 lncRNA genes [12], 20,000–25,000 protein-coding genes [13] and about 2,500 miRNAs [14]. Many of lncRNAs show spatial-temporal and tissue-specific patterns of expression, suggesting thereby that the expression of lncRNAs is programmed and highly regulated during both development as well as pathogenesis. One clue has been their responsiveness to well-characterized transcription factors. In 2003 Martone *et al*. reported the presence of NF-κB-binding sites distributed across the human genome in both coding as well as noncoding regions, thereby suggesting that in addition to mRNA, non-coding RNAs may also be regulated by NF-κB [15]. Indeed, data from Lander's group have shown that lncRNAs are transcriptionally regulated by key transcription factors such as p53, NF-κB, Sox2, Oct4 and Nanog under various external stimuli [16]. For example, in this particular study the authors exposed mouse embryonic fibroblasts (MEF; p53+/+ and p53−/−) to a DNA damaging agent and profiled the cells for changes in lncRNA expression by microarray. Their findings identified 39 lncRNAs that were significantly increased in p53+/+ but not in p53−/− cells. Furthermore, the promoters of these upregulated lncRNAs were found to be enriched for the p53 *cis*-regulatory binding element. These findings thus implicated the role of p53 in transcriptional regulation of lnRNA via direct binding of the transcription factor to the lnRNA promoter. These authors also explored the role of the TLR4/NF-κB signaling pathway in the transcriptional regulation of lncRNA by stimulating CD11c+ bone-marrow-derived dendritic cells with a specific agonist of TLR4. Herein they found marked upregulation of twenty lncRNAs following stimulation including *lincRNA-Cox2* that exhibited ~1000-fold increase. Subsequent studies have further demonstrated that NF-κB-induced *lincRNA-Cox2* also serves as a coactivator of transcriptional factors regulating the expression of a vast array of immune related genes in macrophages, microglia and epithelial cells [17, 18]. In another report, in loss-of-function studies using the pluripotency-associated transcription factors, it was also shown that lncRNA genes were regulated by key transcription factors. In addition, lncRNA expression was also affected in response to many external stimuli. For example, lncRNA *BACE1-AS*, the BACE1-antisense transcript, is upregulated by various cell stressors, such as high temperature, serum, starvation, Aβ1-42 (beta-amyloid 42), hydrogen peroxide (H_2_O_2_) or high glucose [19]. Observations from different groups have demonstrated that lncRNA *Sat III RNAs* are also induced by a wide range of stressors including DNA damaging agents, oxidative stress, hypoxia, hyper-osmotic stress and heavy metals [20, 21]. Additionally, in various tumor cell lines and following DNA damage, induced p53 can bind to the promoter of *lncRNA-p21*, a transcriptional target of p53, leading, in turn, to enhanced expression of the *lncRNA-p21*. Taken together, there is ample evidence demonstrating that similar to mRNA, lncRNA transcription is also under strict regulation by various cellular pathways.

## MOLECULAR MECHANISM(S) UNDERLYING LNCRNA ACTION

**Figure 1:**
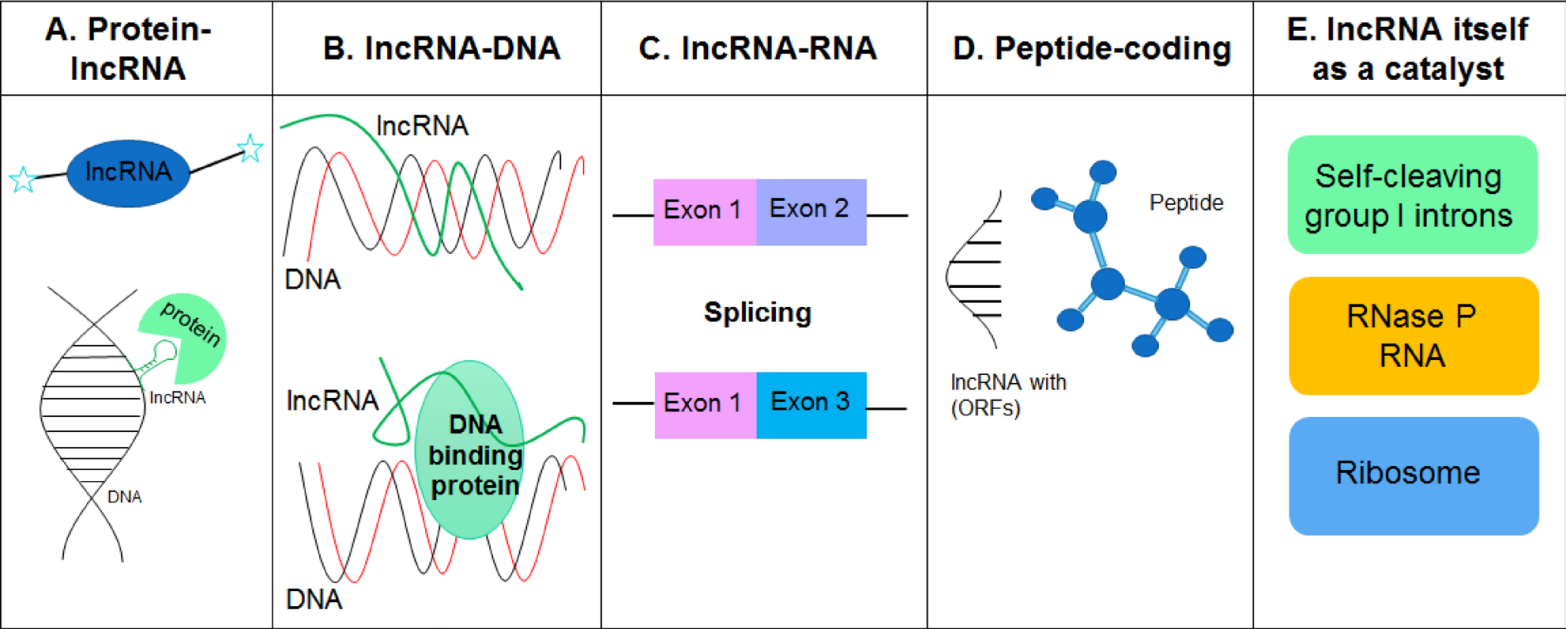
LncRNAs interact with other molecules such as proteins, DNA, RNA and metal ions to form proper tertiary structures (RNA is negatively charged) and exert their functions (**A**) LncRNAs can display complex secondary and tertiary structures that offer multiple binding sites for other molecules. (**B**) LncRNAs can bind to genomic DNA elements, such as gene promoter regions, and regulate gene transcription. (**C**) LncRNAs can bind to other RNAs and are involved in the regulation of mRNA splicing, editing, subcellular distribution and stability. (**D**) LncRNAs with short open reading frames (ORFs) code peptides. Both mRNAs and lncRNAs play dual roles as protein/peptide-coding and non-coding RNAs. (**E**) Catalytic RNAs (ribozyme) such as self-cleaving group 1 introns, RNase P RNA, and the ribosome, play an important role in various biological processes including RNA processing and protein synthesis.

Though little is known about the three-dimensional structures of lncRNAs, it is becoming clear that lncRNAs interact with other molecules, such as proteins, DNA, RNA and metal ions, to form proper tertiary structure to exert their functions. The underlying molecular mechanisms by which lncRNAs affect various biological processes, including chromatin organization, epigenetic regulation, gene transcription and translation, RNA turnover and genome defense, have been reviewed extensively [1, 22–25]. Herein we categorize the mechanisms of lncRNA action into five archetypes based on their interaction partners and direct effects (Figure 1).

### Protein-lncRNA

Unlike miRNAs, lncRNAs are able to display complex secondary and tertiary structures which provide multiple binding sites for other molecules. It is well known that lncRNAs can serve as structural scaffolds to build protein complexes. An excellent example is *HOTAIR* lncRNA that has two arms, each of which binds to a distinct protein, PRC2 and LSD1/coREST/REST, and acts as a scaffold to coordinate the recruitment of protein complexes onto chromatin [26]. The 5′ 300 nt of the *HOTAIR* lncRNA binds to PRC2 while the 3′ 700 nt region interacts with the LSD1/coREST/REST complexes which, in turn, methylates histone H3 at K27 and demethylates histone H3 at K4 to repress gene transcription [26, 27]. Similarly, *ANRIL*, a 3.8 kb-antisense lncRNA, can directly interact with components from both PRC1 and PRC2 complexes and regulate the expression of protein-coding genes in a cis manner at the level of chromatin modification [28, 29]. Interestingly, *Xist*, a 17-kb lncRNA, can also recruit PRC1 and PRC2 to the chromatin [30, 31]. These observations have led to queries over whether lncRNAs interact with each other within the same complex to form a higher order structure and function jointly, and/or whether the lncRNAs play a distinct role in various physiological and pathological conditions. Work in this area warrants further investigations.

### lncRNA-DNA

LncRNAs can also directly bind to genomic DNA elements, such as the gene promoters and regulate gene transcription. In fact, it has been shown that a subset of lncRNAs do contain 5′ boundaries of the protein-coding genes but not complete exons. These lncRNAs are also termed as promoter-associated lRNAs (*PALRs*) [32]. An elegant study by Blume *et al.* demonstrated a nucleus enriched lncRNA, *dhfr* minor transcript, transcribed from the upstream minor promoter of the dihydrofolate reductase (*DHFR*) gene [33–35]. Furthermore, it was also shown that the *dhfr* minor transcript functions as a regulatory molecule to modulate the transcriptional activity of the *dhfr* core promoter [33]. A subsequent study by Martianov and coworkers demonstrated that this lncRNA mediated the formation of a stable RNA-DNA triplex structure within the major promoter of *DHFR* gene that prevented the binding of the general transcription factor TFIIB, thereby leading to repression of the DHFR gene expression [36]. A standard *H-form* band-shift assay further demonstrated that the formation of the lncRNA-DNA complex yielded a highly specific and stable triplex structure [36]. In addition to RNA-DNA triplex structure, lncRNAs can also form duplexes with DNA elements and establish complex configurations based on sequence complementarity [37]. For example, lncRNA *ANRASSF1* (antisense noncoding of *RASSF1)* recruits PRC2 to the *RASSF1A* promoter region, where both the lncRNA and the host gene are transcribed in a highly location-specific manner leading, in turn, to repression of *RASSF1A* transcription [38]. The direct interaction between *ANRASSF1* and promoter was also evidenced by a reduced level of *ANRASSF1* in cell lysis treated with RNase H, that digests RNA/DNA hybrids [38]. Further research is needed to understand: a) the multitude of components comprising the lncRNA/DNA complex, b) whether lncRNAs exert their function at the local genome loci during or subsequent to their production and, c) the fate of these lncRNAs.

### lncRNA-RNA

Within the nucleic acid structure of lncRNAs lies their inherent ability to bind to other RNAs, such as mRNAs and miRNAs, with either imperfect or perfect complementarity. By directly interacting with mRNAs, lncRNAs play a key role in the regulation of mRNA splicing, editing, subcellular distribution and stability [37, 39]. For example, Hu and coworkers demonstrated that lncRNA *5S-OT* (transcribed from 5S rDNA loci) modulates alternative splicing of a subset of mRNAs *in trans* via RNA-RNA pairing and by interacting with the splicing factor U2AF65 [39]. Interestingly, *5S-OT* knockdown resulted in altered splicing of about 174 and 173 genes in both undifferentiated and differentiating THP-1 cells, respectively, leading in turn, to decreased differentiation efficiency of THP-1 cells [39]. Additionally, it was shown by Johnsson *et al.* that the *PTEN* pseudogene expressed a noncoding RNA, *PTENpg1*, which serves as a miRNA sponge, can interact with β isoform of its antisense RNA through RNA-RNA pairing. This interaction leads to increased sponge activity of *PTENpg1* sense miRNA thereby resulting in increased *PTEN* mRNA stability and translation [40]. Furthermore, it has been shown that lncRNA *EWSAT1* (Ewing sarcoma associated transcript 1) acts as a reservoir of miR-326/330-5p [41]. Highly expressed *EWSAT1* in human nasopharyngeal carcinoma tissues and cell lines increases the expression of miR-326/330-5p clusters that target the gene cyclin D1, ultimately regulating NPC development and progression [41]. Although several lncRNA-miRNA-mRNA networks have been identified in recent years [42, 43], the underlying mechanism(s) by which miRNA competes with binding either lncRNA or mRNA still remains unclear.

### Peptide-coding

Although by definition, lncRNAs generally lack protein-coding capacity, however, recent studies have demonstrated that a small fraction of lncRNAs with short open reading frames (ORFs) actually codes for peptides with biological functions [44–48]. For example, it was shown by Anderson and colleagues that a putative lncRNA, myoregulin (*MLN*), contains a short ORF encoding a conserved 46 amino acid peptide [45]. The human and mouse *MLN* genes comprising of three exons span the genomic regions of 16.5 and 15.0 kb, respectively. Biochemical and animal studies have indicated that *MLN* forms a single transmembrane alpha helix and interacts with *MLN* in the membrane of the SR (sarcoplasmic reticulum) and plays a role in regulating SR Ca^2+^ levels by inhibiting SERCA pump activity. Strikingly, *MLN* KO mice demonstrated significantly increased SR Ca^2+^ levels in myoblasts with enhanced skeletal muscle performance compared to WT mice, indicating thereby that *MLN* plays an important role in adult skeletal muscle functioning [45]. Similarly, lncRNA *DWORF* (dwarf open reading frame) encodes a 34 amino acid peptide that localizes to the SR membrane [46]. In animal studies, it was shown that *DWORF* overexpression increased the peak systolic Ca^2+^ transient amplitude as well as SR Ca^2+^ load and that, conversely, *Dworf* KO mice showed decreased affinity of SERCA for Ca^2+^ with delayed Ca^2+^ clearance and relaxation [46]. Another lncRNA with a protein coding capacity is *LINC00961* that has been identified as a repressor of mTORC1 activation, via its interaction with the lysosomal v-ATPase in the late endosome/lysosome [44]. *LINC00961* is highly expressed in the lung, heart and skeletal muscle and yields a 90 amino acid polypeptide. Interestingly, in animal studies it was demonstrated that loss of this polypeptide promoted muscle regeneration following injury [44]. These studies thus underscore the fact that peptides encoded by lncRNAs play a key role in the maintenance of muscle homeostasis. It is thus speculated that both mRNAs and lncRNAs can play dual roles as either protein/peptide-coding as well as non-coding RNAs.

### lncRNA as a catalytic RNA

Catalytic RNAs (known as ribozymes) are RNA molecules that have catalytic enzyme activity. Catalytic RNAs, such as the self-cleaving group I introns [49], the RNase P RNA [50, 51] and the ribosome [52], play a role in various biological processes, including RNA processing and protein synthesis. Given that secondary and tertiary structures are important for their function and regulation of catalytic RNAs, such as ribosomal RNA (rRNA) and transfer RNA (tRNA), and the experimental difficulties in measuring RNA structure, computational approaches for predicting RNA structure from primary sequence have been developed and used in various studies. Experimentally, nucleotide analog interference mapping (NAIM) is an efficient and a powerful approach to study RNA substructures and functional groups at the atomic level. This method has been applied to identify many catalytic RNAs, such as the Hepatitis Delta Virus (HDV), the hairpin and the Varkud Satellite (VS) ribozymes [53]. Thus, within the lncRNA community efforts aimed at combining computational and experimental approaches to reveal the catalytic functions of lncRNAs and their underlying mechanisms are gaining a lot of momentum.

In summary, lncRNAs participate in the regulation of gene expression at many levels, including epigenetic, transcriptional, RNA splicing, nuclear shuttling, post-transcriptional, translational and post-translational, and are mediated by various underlying mechanisms.

## LNCRNAS IN HUMAN DISEASES

The role of lncRNAs in the regulation of cellular processes and disease progression is gaining attention. While lncRNAs are known to play pivotal roles in maintaining cellular and organismal homeostasis, dysregulation of their expression during the onset and development of various diseases has often been implicated in the regulation of a wide array of genes including those involved in metabolism, cell death, angiogenesis and metastasis. LncRNA-mediated regulation of disease pathogenesis (Table 1) thus represents an evolving area of research that has ramifications for the identification of potential therapeutic targets for various diseases, including cancers and neurodegenerative disorders, for which, currently there exists no cure. Several elegant reviews have recently highlighted and described the role of lncRNAs in colorectal cancer [54, 55], prostate cancer [56–58] and rheumatic diseases [59]. Herein, we briefly summarize the latest findings on the role of lncRNAs in breast cancer, bladder cancer, cardiovascular and CNS diseases.

**Table 1:** LncRNA-mediated regulation of disease pathogenesis

Disease	LncRNAs	Target	Role of LncRNAs	Model	Reference
Alzheimer's Disease (AD)	UP: BACE 1 – AS	UP: BACE1	Increase BACE1 mRNA stability and generate additional Aβ1-42	Amyloid precursor protein transgenic mice, BACE1-AS Knock down in Tg 19959 mouse	[19, 100]
	UP: 51A	Down: SORL1 variant A	Impaired processing of APP and increased Aβ formation	Frontal and temporal cortices from AD and control cases, Neuroblastoma cell lines (SKNBE2 and SHSY5Y)	[101]
	Up: 17A	Abolish GABA B2 intracellular signaling	Enhances Aβ secretion and Aβ x-42/ Aβ x-40 ratio	Frontal and temporal cortices from AD and control cases, neuroblastoma cells	[145]
	Up: NDM29	Induce APP synthesis	Enhances Aβ secretion and Aβ x-42/ Aβ x-40 ratio	Frontal and temporal cortices from AD and control cases, neuroblastoma cells	[146]
	Up: BC200	Inhibit eIF4A	Repress local translation in synapses	Brodmann's area 9 in AD and different ages of human cases	[107]
	Up: NAT-Rad18	RAD18	Cause Neuron more sensitive to apoptosis	beta-amyloid (Abeta) exposed protein rat cortical neurons	[147, 148]
Huntington Disease (HD)	Down: HAR1F Down: HAR1R		associated with human-specific brain development and function, repressed by REST in HD	Normal and HD subjects’ cortex (Brodman Area 7, 9) and striatum	[118]
	Down: ABHD11OS	N-terminal fragment of mutant huntingtin	Product neuroprotection	BACHD Tg and KI140 mouse	[120]
	Down: TUNA	TUNA-RBP complex	Inhibits neural differentiation of mESCs	mouse embryonic stem cells, zebrafish	[119]
Parkinson disease (PD)	PINK1-AS (naPINK 1)	PINK1	Impairment of mitochondrial dynamics due to decrease in the PINK1-AS and neurodegeneration due to ASUCHL1 downregulation	Human muscle biopsy samples, neuronal cell lines	[127]
	UP: HOTAIR	LRRK2	Promote Parkinson's Disease induced by MPTP	Neurochemical mouse model of PD	[149]
	Down: AS Uch1	Uchl1	Controlled by Nurr1, a transcription factor required for DA cells differentiation	Murine dopaminergic MN9D cells, Neurochemical mouse model of PD	[131]
Cardio Vascular disease (CVD)	CHAER	Catalytic subunit of polycomb repressor complex 2 (PRC2)	Gene induction and hypertrophy of cardiac muscles	CHAER knock out mouse model	[98]
	Up: MALAT1	miR133	increase levels of serum response factor (SRF)	α-MHC-SRF transgenic mouse	[150] [151]
	Up: KCNQ1OT1	KCNQ1	Affects chromatin conformation and expression of Kcnq1	K-term mouse	[150, 152]
	Down: Novlnc6	Bmp10 and Nkx2.5	Involved in cardiogenesis, also have essential functions in the progression of acute myocardial infarction	Plasma from CAD patients, VSMCs	[153, 154]
	Down: Mhrt	Brg1	cardiac hypertrophy and subsequent heart failure	Tnnt2-rtTA, Tre-Mhrt779 (Tg779) mice, human heart tissue	[97]
Breast cancer	Up: H19	miR-200b/c and let-7b	Regulates the metastasis	DSCAM- AS1 knock down in luminal breast cancer cell lines	[155]
	Down: XIST	AKT	XIST negatively regulates cell viability via inhibition of AKT activation	Breast cancer cell line	[156]
	Up: MALAT1	Interacts with estrogen receptor, serine/arginine splicing factors	regulate process of cancer cell migration, cell cycle progression and alternative splicing	Breast cancer cell lines, non-metastatic breast tumors patients, MMTV-PyMT Malat1+/+; MMTV-PyMT Malat1+/−; MMTV-PyMT Malat1−/− mice	[92, 157, 158]
	Up: DSCAM-AS1	hnRNPL	mediates tumour progression and tamoxifen resistance	T47D, ZR75-1, MCF7 cells; human breast cancer	[70]
Bladder cancer	Down: TUG1	HMGB1	Potential regulator of radioresistence of bladder cancer	siRNA knock down of TUG1	[160]
	Up: UCA1	Hexokinase 2	Promotes glycolysis	human bladder cancer cell lines	[159]

### Role of lncRNAs in breast cancer

The lifetime risk of breast cancer is about 1 in 8 for all women with 11% mortality from the disease within five years of diagnosis. Since the presence of hormone receptors, such as the estrogen receptor (ER), progesterone receptor (PR) and human epidermal receptor 2 (HER2) are critical for cancer cell growth, these receptors are often used for diagnosis and as therapeutic targets to treatment of breast cancer.

The earliest identified and most extensively studied cancer-related lncRNAs include *H19*, *XIST* and *MALAT1*. *H19* was first identified as imprinted fetal lncRNA that was highly expressed in ER+ breast tumors and cell lines. Interestingly, *H19* was also increased in paclitaxel (PTX, often used post-surgery to kill remaining cancer cells) resistant ER+ breast cancer cells. *H19* promotes proliferation and attenuates apoptosis in breast cancer cells, implicating its role in tumorigenesis and tumor growth [60–64]. In contrast, *XIST* is a downregulated lncRNA found in breast tumor samples and other cancer cell lines that acts as a tumor suppressor. Loss- and gain-of-function studies demonstrated that *XIST* negatively regulates breast cancer cell viability through suppression of AKT signaling. *MALAT1* is significantly upregulated in breast cancer, especially in ER+ and HER2+ tumors, and *MALAT1* is required for breast cell proliferation, migration and invasion, as knockdown of *MALAT1* results in slower tumor growth and a reduction in metastasis, indicating its role in cancer development and progression [65–69].

To comprehensively identify breast cancer-associated lncRNAs, Niknafs and co-workers investigated lncRNA expression profiles from 947 breast tumor RNA-seq samples and demonstrated that almost 437 of the lncRNAs were differentially expressed in breast cancer [70]. Among these, 63 lncRNAs were upregulated in both breast cancer cells compared with normal and ER+ compared with ER- tumors, including *DSCAM-AS1*. Indeed, *DSCAM-AS1* is a cancer specific lncRNA that is highly expressed in ER+ cells, and is involved in the proliferation of luminal breast cancer cell lines [71]. *DSCAM-AS1* knockdown reduced the proliferative ability of breast cancer cell lines while *DSCAM-AS1* overexpression enhanced proliferation of ER+ breast cancer cell lines [70]. Importantly, in tamoxifen-resistant cells, the expression of *DSCAM-AS1* was further upregulated, despite reduced expression of canonical ER targets (GREB1 and PGR) compared to the parental cells. Furthermore, knockdown of *DSCAM-AS1* in these tamoxifen-resistant cells reduced the proliferation rate to the level of parental MCF7 cells, suggesting a role of *DSCAM-AS1* in tumor progression and tamoxifen resistance in ER+ breast cancer [70, 71].

Recently, another comprehensive analysis was performed by Van Grembergen and colleagues to explore the lncRNA landscape across 995 breast tissue samples [72]. Using Affymetrix Human Genome U133 Plus 2.0 array data from 823 breast tumors and 172 normal breast tissues, 215 lncRNAs were identified as dysregulated in at least 10 % of the breast tumors. In terms of function, these lncRNAs were found to underlie various molecular processes, including the EGFR, PI3K, MAPK and E2F1 pathways. Specifically, a set of lncRNAs were differentially expressed in ER+ and ER– breast tumors, including well-known ones such as *NEAT1*, *MALAT1* and *Xist*, and also novel lncRNAs *LINC01297* and *RP11-303E16.2*. Of note, lncRNA-*CYTOR* (cytoskeleton regulator, also known as LINC00152) was up-regulated in all subtypes of breast cancer and was found to functionally regulate cell proliferation, migration and cytoskeleton organization [72]. The role of lncRNAs in breast cancer has recently been insightfully reviewed by Soudyab *et al.* [73] and by Warburton and Boone [74].

### Role of lncRNAs in bladder cancer

Bladder cancer usually arises from the epithelial cell lining of the bladder. Cystoscopy is a gold standard for diagnosing bladder cancer, albeit it often comes with the risk of infection or injury to the bladder. Detection of genetic aberrations with fluorescent *in situ* hybridization in the cells that are shed in urine is a widely-used non-invasive method for the diagnosis of bladder cancer; however, it is more expensive than cystoscopy and has lower sensitivity and specificity. There is therefore an urgent need to identify novel biomarkers for early detection and routine screening for bladder cancer. Emerging evidence demonstrates that lncRNAs play a pivotal role in bladder cancer initiation, development and metastasis, thereby underpinning their potential roles as novel indicators of bladder cancer as well as therapeutic targets. To date, many aberrantly expressed lncRNAs have been identified in bladder cancer, including upregulated lncRNAs, *H19*, *MALAT1*, *SNHG16*, *TUG1*, *UCA1*, *TINCR* and *Linc-UBC1*; and downregulated lncRNAs, *BANCR* and *MEG3* [75–78]. *H19* was the first lncRNA to be associated with bladder cancer [79] and its expression is significantly increased in bladder cancer tissue versus the adjacent normal control tissue [80, 81]. Further studies demonstrated that upregulated *H19* promoted proliferation and metastasis of bladder cancer cells via upregulation of inhibitor of DNA binding/differentiation 2 (ID2) and downregulation of E-cadherin [81, 82]. Urothelial cancer associated 1 (UCA1) lncRNA is highly expressed in bladder tumor tissues [83–85] and has been used to distinguish bladder cancer from other urinary related diseases [86]. Both *in vitro* and *in vivo* studies demonstrated that overexpression of *UCA1* increased proliferation, migration, invasion and chemoresistance of bladder cells; antagonized cell apoptosis induced by cisplatin and promoted tumorigenicity of human bladder carcinoma cells [87, 88]. Recent studies have suggested that CCAAT/enhancer binding protein alpha (C/EBP alpha) positively regulates *UCA1* expression. Upregulated *UCA1* resulted in increased Wnt signaling and enhanced ERK1/2 MAPK and PI3-K/AKT kinase activity and p300 expression which, in turn, regulated gene transcription via chromatin remodeling that resulted in cell cycle progression, carcinogenesis and cancer invasion [89, 90]. In addition, *TUG1* is another lncRNA that was significantly increased in bladder cancer and was associated with reduced overall survival of bladder cancer patients [91]. Results from *in vitro* studies demonstrated that knockdown of *TUG1* by siRNA significantly decreased proliferation and migration of bladder cancer cells [91], implicating thereby that lncRNAs could also be envisioned as potential therapeutic targets for preventing cancer invasion and metastasis.

**Figure 2:**
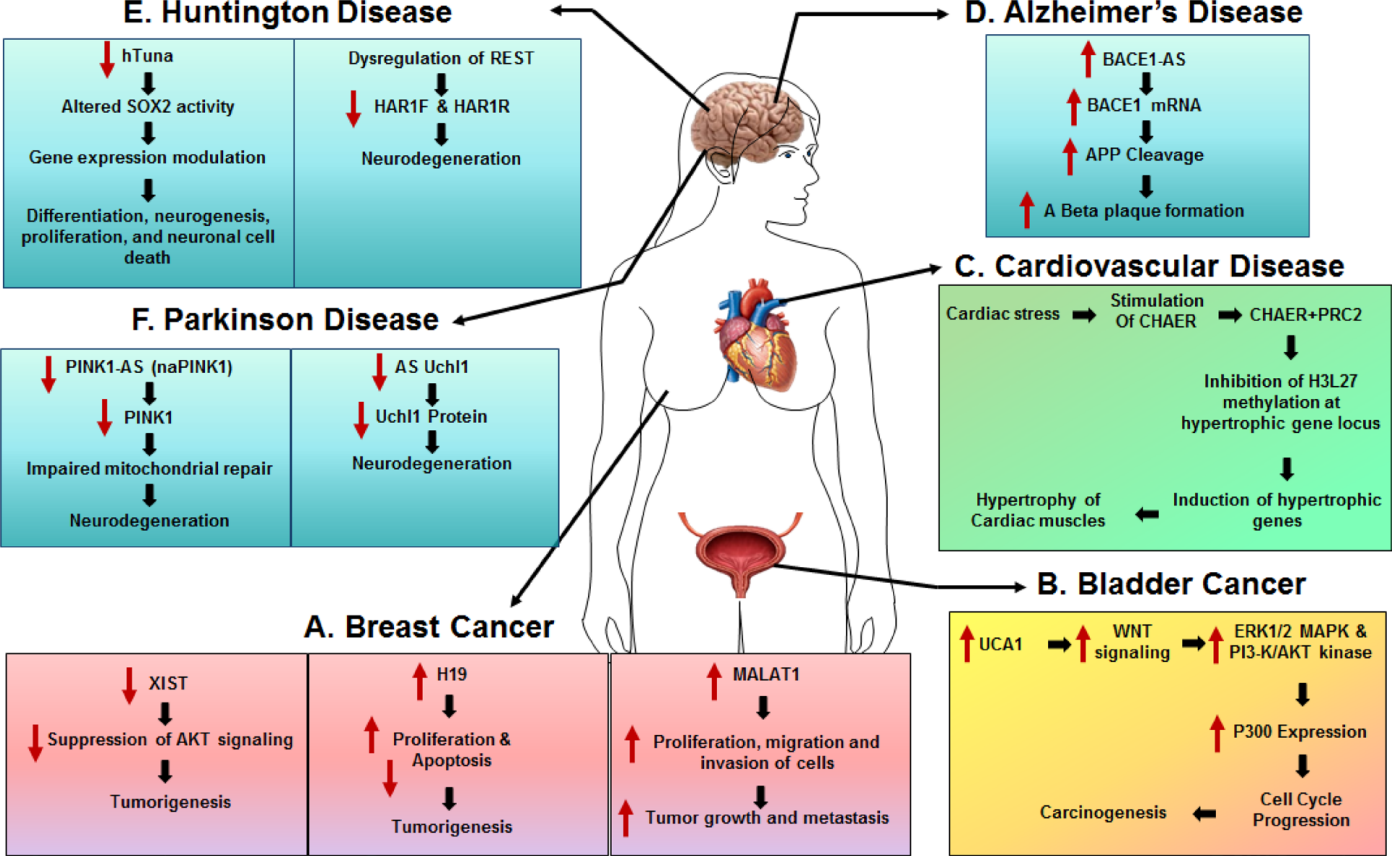
The role of lncRNAs in human diseases (**A**) Breast Cancer. LncRNAs, H19 and MALAT1 are both significantly upregulated in breast cancer and are involved in tumorigenesis and tumor growth. LncRNA XIST is downregulated in breast tumor and acts as a tumor suppressor via regulating AKT signaling. (**B**) Bladder Cancer. LncRNA UCA1 is highly expressed in bladder tumor tissues and promotes cell growth and tumorigenesis. (**C**) Cardiovascular Disease. Heart-enriched lncRNA, Chaer is upregulated by cardiac stress and is involved in development of cardiac hypertrophy. (**D**) Alzheimer's Disease. Upregulated lncRNA BACE1-AS led to a significant increase of BACE1 mRNA in AD brains, subsequently exacerbating Aβ plaque formation.(**E**) Huntington Disease. Human lncRNA hTUNA negatively correlates with severity of Huntington Disease by regulating SOX2 activity. (**F**) Parkinson Disease. LncRNAs PINK-As and AS Uch1 negatively correlate with severity of Huntington Disease

Though there is no evidence supporting the fact that any single lncRNA could serve as a reliable biomarker for cancer, multiple lncRNAs (together as a panel) could be used for cancer diagnosis at various stages, especially, given that distinct lncRNAs could be dysregulated by various risk factors leading to oncogenesis (Figure 2).

### Role of lncRNAs in cardiovascular disease (CVD)

Cardiovascular disease (CVD) is a pathological spectrum involving dysfunction of the heart and/or blood vessels. Many risk factors have been identified for CVDs, including age, gender, diet, environment and genetics. Among these, genetic factors play a major role in the development of CVDs. Given that 98% of the human genome is transcribed into lncRNAs [92–94], it is not surprising that lncRNAs are involved in cardiac development and disease. For example, in a pressure-overload-induced heart failure mouse model, Lee and colleagues demonstrated that almost 150 out of the 700 assessed lncRNAs were differentially expressed during various stages of heart failure [95]. Importantly, Yang *et al.* detected 18,480 lncRNAs in the human left ventricle using RNA sequencing. Out of these, about 1,249 lncRNAs were differentially expressed in heart failure [96]. Further analyses implicate that cardiac lncRNAs could act through cis mechanisms in mediating the pathogenesis of heart failure [96].

In another study performed by Han and colleagues, a cluster of lncRNAs transcribed from the Myh7 loci designated as myosin heavy-chain-associated RNA transcripts (*Myheart*, or *Mhrt*) [97], was identified in mice. Interestingly, these lncRNAs that are cardiac-specific with high abundance in the adult heart were found to be repressed in various types of cardiac myopathies. In this study, the authors demonstrated that overexpression of *Mhrt* resulted in reduced hypertrophy with improved fractional shortening in transaortic constricted (TAC) mice. The cardioprotective effects of *Mhrt* suggested a potential role of lncRNAs as therapeutics for CVDs. A subsequent study identified another heart-enriched lncRNA, cardiac-hypertrophy-associated epigenetic regulator (*Chaer*), that is required for the development of cardiac hypertrophy [98] and that has been shown to play a role in regulating cardiomyocte gene expression in both rodents and humans [98]. It must be noted that most of the findings on cardiac lncRNAs have been reported in either cell culture or in animal models with scant information in humans. Understanding how these animal studies translate into the human context is an urgent need in the field.

### Role of lncRNAs in Alzheimer's disease (AD)

Alzheimer's disease (AD) is the most common cause of dementia (a group of brain disorders) often manifesting in late life, that is clinically characterized by loss of intellectual and social skills. The AD brain shows increased accumulation of extraneuronal amyloid beta (Aβ) plaques and intraneuronal aggregation of hyperphosphorylated tau in neurofibrillary tangles with various other accompanying pathological features such as microglial activation and neuronal degeneration. Aβ is generated from sequential cleavage of amyloid precursor protein (APP) by β-site APP-cleaving enzyme 1 (BACE). LncRNA, *BACE1-AS*, an antisense transcript of *BACE*, has been shown to be increased in the human AD brains compared with the matched controls [19]. RT-PCR analysis demonstrated that *BACE1-AS* transcripts are two to three times more abundant in glial cells (M059K), a major source of A*β* production in the brain [99], compared with human cortical neurons (HCN1A). Interestingly, upregulated *BACE1-AS* leads to a significant increase in *BACE1* mRNA and rapid feed-forward regulation of *β*-secretase, implicating the role of *BACE1-AS* in driving AD pathology [19]. RNase protection assay (RPA) further demonstrated that *BACE1* and *BACE1*-AS can form an RNA duplex that stabilizes the complex, protecting it from degradation [19]. Additionally, evidence from a study by Modarresi *et al.* has demonstrated that knockdown of *BACE1- AS* in the brains of Tg19959 mouse (a mouse model of AD) modulates A*β* related hippocampal neurogenesis [100]. Another transcript increased in the AD brains, is the *lncRNA 51A* that is an AS transcript of the AD associated gene, sortilin-related receptor 1 (*SORL1*). Its upregulation is associated with an alternative splicing of *SORL1,* leading to impaired secretion of Aβ [101].

Glial cell-derived neurotrophic factor (GDNF) is an essential neurotrophic factor that is downregulated in both aging and AD. *GDNFOS1* and *GDNFOS2* are complementary non-protein coding transcripts of the human *GDNF* gene [102]. Dysregulated expression of *GDNFOS1* and *GDNFOS2* in AD brains has been speculated to contribute to AD progression and pathogenesis [102, 103]. LncRNA, *BC200 RNA* (also known as *BCYRN1*) can regulate gene expression at the translational level [104–106]. Interestingly, there exists a close parallel between relative levels of *BC200 RNA* in the affected brain areas and the progression of AD, as determined by clinical dementia rating scores. Increased perikaryal accumulation of *BC200* during advanced stages of the disease likely suggests that aberrant localization and overexpression of *BC200* could contribute to synaptodendritic deterioration observed in AD [107].

Additionally, single-nucleotide polymorphisms (SNPs) within the human genome have been associated with various human diseases. Recent studies have shown that SNPs (rs3217992; rs1063192, and rs1333049) in lncRNA, *ANRIL* (also known as *CDKN2BAS* or *CDKN2B-AS1*) are linked to various physiological and pathological states, such as cardiovascular disease, type 2 diabetes and AD [108–111]. Furthermore, by reannotation of the microarray data, Zhou and Xu have identified 24 upregulated and 84 downregulated lncRNAs in AD patients compared with controls [112]. The highly upregulated lncRNA, *n336934* (GenBank: BC017047) was found to be encoded by the mitochondrial genome and associated with cholesterol homeostasis pathway, that plays a pivotal role in regulating Aβ formation in AD [113]. Given that the mitochondrial genome is exclusively maternally inherited in humans, this mitochondrial lncRNA could play an important role in maternally inherited AD [114].

To date, many lncRNAs have been associated with AD and regulate the expression of AD-associated genes; however, the mechanisms by which lncRNAs affect AD onset, progression and pathogenesis require further investigation. Thus, lncRNAs hold the potential to be used as biomarkers for AD development and could serve as targets for therapy.

### Role of lncRNAs in Huntington's disease (HD)

Huntington's disease (HD; also known as Huntington's chorea) is an inherited progressive degenerative disorder that develops early in life (40 s or younger). HD results in the death of nerve cells in the brain, leading to movement, cognitive and psychiatric disorders [115]. Available treatments can relieve the symptoms of HD, but do not provide a cure [116]. Human accelerated regions (HARs) represent evolutionary conserved segments of the human genome. Several of the *HARs,* including lncRNAs, *HAR1F* and *HAR1R,* are involved in transcriptional regulation and play an important role in neurodevelopmental processes [117]. In fact, both *HAR1F* and *HAR1R* are significantly downregulated in the striatum of HD patients compared with unaffected individuals [118]. A ChIP-seq (chromatin immunoprecipitation-sequencing) study revealed that *HAR1F* and *HAR1R* are regulated by the transcriptional repressor REST (RE1-silencing transcription factor) and dysregulation of these lncRNAs contributes to neurodegeneration in HD [117]. Similarly, human lncRNA, *hTUNA* negatively correlates with disease severity in the caudate nucleus of HD brains [119]. At the molecular level, by its interaction with the RNA-Binding Proteins PTBP1, hnRNP-K and Nucleolin, *T*UNA regulates Sox2 activity. Both TUNA and Sox2 modulate the expression of 562 genes involved in development, differentiation, neurogenesis, proliferation and neuronal death, indicating the role of lncRNAs in HD progression [119]. Interestingly, lncRNA *Abhd11os* (known as *ABHD11-AS1* in humans) is significantly downregulated in the striatum of HD mouse models [120], whereas, forced overexpression of *Abhd11os* ameliorates neurotoxicity in HD mice, thereby underscoring the role of lncRNAs as potential novel therapeutic targets for HD. Importantly, microarray data from the caudate nucleus of HD brains demonstrated upregulation of 35 lncRNAs and downregulation of 146 lncRNAs compared to matched controls [121]. NEAT1, one of the upregulated lncRNAs in HD [122, 123], showed protective effect against oxidative injury in neuronal cell lines [121]. Although the role of HD-associated lncRNAs is still poorly understood, elucidating lncRNA expression patterns and regulating networks in HD could provide biologic predictors of disease severity, while also shedding light on therapeutic strategies.

### Role of lncRNAs in Parkinson's disease (PD)

Parkinson's disease (PD) is a progressive movement disorder resulting from the loss of dopaminergic neurons in the substantia nigra of the human brain. Currently there is neither a cure nor an exact underlying cause(s) known for PD. Many PD-related genes are associated with mitochondrial function, such as alpha-synuclein, Parkin, PINK1 (phosphatase and tensin homologue induced putative kinase 1), Ubiquitin carboxy-terminal hydrolase L1 (Uchl1), DJ-1 (also known as PD protein 7, PARK7) and LRRK2 (leucine-rich repeat kinase 2), indicating thereby that mitochondrial dysfunction could play a central role in PD pathogenesis [124–126]. PINK1 is a mitochondrial kinase and an upstream factor of Parkin. PINK1 is located in the inner mitochondrial membrane and acts as a recruiter of Parkin to the mitochondria following mitochondrial depolarization. Both proteins together are important for governing mitochondrial dynamics and quality control. In a study Scheele *et al.* discovered a non-coding antisense transcript expressed at the human PINK1 locus, *naPINK1* (also known as *PINK1-AS*), that can positively regulate a cis-transcribed mRNA - a novel splice variant of PINK1 (*svPINK1*) which is homologous to the C-terminus regulatory domain of the protein kinase [127]. Northern blotting analysis further demonstrated the expression of 4.4 kb *naPINK1* in 4 neuroblastoma cell lines, including SH-SY5Y, SK-N-SH, SK-N-AS and SK-N-F1 [127]. Similarly, Chiba *et al.* reported enrichment of *naPINK1* and *PINK1* mRNA in the hippocampus of newborn and 1-week-old mice using *in situ* hybridization [128]. The role of *naPINK1* in mitochondrial dysfunction and PD pathogenesis however, remains to be investigated [129].

In another study by Carrieri *et al.* it was shown that lncRNA *AS Uchl1* (also known as *UCH1LAS*), itself an antisense RNA to the mouse *Uchl1* gene, was enriched in the nucleus of dopaminergic neurons [130] and was found to increase UCHL1 protein synthesis at the post-transcriptional level. Importantly, *AS Uchl1* was significantly downregulated in DA neurons purified from 1-methyl-4-phenyl-1, 2, 3, 6-tetrahydropyridine (MPTP) injected mice (a mouse model of PD) [131]. Since UCHL1 protein has been shown to contribute to the progression of neurodegenerative diseases, *AS Uchl1*, as its upstream regulator, could be envisioned as a new therapeutic target.

In their efforts to discover novel blood-based diagnostic biomarkers for PD, Soreq *et al.* using RNA-seq and comprehensive computational workflow, identified 7,000 brain-expressed lncRNAs, among which 3,495 lncRNAs were also co-expressed in the leukocytes [132]. Of interest, five of these lncRNAs were elevated in PD patients’ leukocytes compared to controls and inversely, were decreased following post-deep brain stimulation (DBS). DBS ameliorated PD-induced expression of *RP11-79P5.3* in both leukocytes as well as two PD brain regions - the amygdala and substantia nigra, compared with controls.

Additionally, *U1*- the spliceosome component as well as *RP11-462G22.1* (also known as *lnc-FRG1-3* and the muscular dystrophy-associated lncRNA) were increased in both the brain and leukocytes of PD patients, suggesting that lncRNAs could also contribute to splicing modulations and muscle rigidity in PD. Although lncRNAs are enriched in the CNS and are dysregulated in various CNS disorders, including PD, the role of lncRNAs in PD pathogenesis warrants further investigation.

### Role of lncRNAs in Prader–Willi syndrome (PWS)

Prader–Willi syndrome (PWS) is a genetic and neurodevelopmental disorder characterized by retarded growth, obesity, muscular hypotonia and mental deficiency [133]. PWS is caused by the loss of function of specific genes in a particular region of chromosome 15q11-q13. Transcripts from this region are associated with this rare disease. These transcripts include protein-coding mRNAs, such as *Ndn* (*Necdin*) and *MAGEL2*, as well as lncRNAs, such as the class of lncRNAs derived from the *SNORD116* snoRNA cluster [134–137]. Yin *et al.* identified a class of lncRNAs, named *sno-lncRNA1-5*, that are processed on both ends by the snoRNA machinery [134]. Northern blotting analysis demonstrated that *sno-lncRNAs* are expressed in many different cell types, including pluripotent cells such as human ES H9 cells and ovarian carcinoma PA1 cells, but not in HeLa cells [134]. Intriguingly, all five PWS region sno-lncRNAs are retained in the nucleus, co-localize, and accumulate at or near their site of transcription. Crosslinking and immunoprecipitation followed by deep sequencing from human embryonic stem cells using antibodies to Fox2 demonstrated that sno-lncRNAs are strongly associated with the splicing factor Fox2. This study thus suggested that one or more of the sno-lncRNAs from the PWS region could contribute to the pathogenesis of this disease by acting as a molecular sink for Fox proteins [134]. Another lncRNA transcribed from *SNORD116* snoRNA cluster, *116HG* can bind to RBBP5 and to the gene promoters genome-wide and form RNA cloud nuclear domains in both mouse and human adult neuronal nuclei [138]. Furthermore, *116HG* regulates the diurnal energy expenditure of the brain which provides a novel mechanism underlying sleep problems in PWS patients [138]. In contrast to *116HG*, lncRNA, *IPW* (Imprinted gene in the Prader-Willi syndrome region), is expressed in the cytoplasm [139]. As *IPW* is located in the 15q11-q13 region, PWS patients do not express *IPW* [139]. Recently, a study of atypical submicroscopic 15q11-q13 deletions in five PWS patients demonstrated a critical deletion region involving the non-coding snoRNA *SNORD116*, *SNORD109A* and *IPW* that was common in all the five individuals, further supporting the role of these lncRNAs in the pathogenesis of PWS [140].

### Role of lncRNAs in fragile X syndrome (FXS)

Fragile X syndrome (FXS) is a genetic disorder caused by a mutation of the fragile X mental retardation 1 (*FMR1*) gene on the X chromosome. It is typically due to an increase in the number of CGG trinucleotide repeats in the 5’ untranslated region of *FMR1*. The abnormally expanded CGG segment results in silencing of the *FMR1* gene with subsequent abnormalities in the formation and function of synapses [141]. Intriguingly, several lncRNAs, including *FMR4*, *FMR5*, *FMR6* and *FMR1-AS*, are transcribed from the *FMR1* gene locus as well [142]. Using gene expression microarrays, Peschansky *et al.* identified a wide array of *FMR4*-responsive genes, suggesting a broad role of *FMR4* [143]. Further analysis confirmed that *FMR4* was involved in cell proliferation, differentiation as well as apoptosis [143]. Using Deep-RACE (rapid amplification of cDNA ends) with sequencing, Pastori *et al.* discovered two lncRNAs, *FMR5* and *FMR6* [144] that are transcribed upstream of the *FMR1* promoter, and antisense of the 3′ region of *FMR1*, respectively. RT-PCR analysis demonstrated that *FMR5* and *FMR6* are expressed in most adult brain regions, including the adult human cerebellum, frontal and temporal lobes, occipital and cerebral cortices and hippocampii, as well as in the fetal brain [144]. The expression of *FMR6* was found to be significantly decreased in the brain of FXS individuals compared with that of the controls [144]. The expression of *FMR5* however, remained unchanged in the brains of FXS individuals [144]. While the expression pattern of these lncRNAs has been determined, efforts aimed at deciphering their functional properties warrants investigation.

## CHALLENGES AND PERSPECTIVES

Tremendous progress has been made in recent years in understanding the mechanisms of action underlying lncRNAs. LncRNAs not only act as transcriptional and post-transcriptional regulators of gene expression, but are also emerging as catalytic enzymes. The rapidly expanding body of work on *lncRNAs* provides evidence that it is feasible to develop computational models that can successfully predict lncRNA structure and function. Additionally, although many studies have been performed in clinical patients using various disease models, the exact role and impact of lncRNAs in disease pathogenesis still remains obscure. Further efforts are required to understand the impact of lncRNAs in all realms of research including basic, translational, clinical and pharmaceutical sciences with specific emphasis on their role in genetic, metabolic, infectious, cancer and age-related diseases.
